# Supplementary material for: MetaQTL: a package of new computational methods for the meta-analysis of QTL mapping experiments
Source: BMC Bioinformatics. 2007 Feb 8;8:49. doi: 10.1186/1471-2105-8-49 (PMC1808479; doi:10.1186/1471-2105-8-49)
Supplement: Additional File 7 — MetaQTL Package : jar file and tutorial. This Zip archive contains both the MetaQTL JAR file and the files of the tutorial. [file 1471-2105-8-49-S7.zip › org.inra.metaqtl/doc/org/metaqtl/ChromCluster.html]

ChromCluster


|  |  |  |
| --- | --- | --- |
| **Overview** \| **Package** \| **Class** \| **Use** \| **Tree** \| **Deprecated** \| **Index** \| **Help** | |  |
| PREV CLASS   **NEXT CLASS** | **FRAMES**    **NO FRAMES**     **All Classes** |  |
| SUMMARY: NESTED \| FIELD \| CONSTR \| METHOD | DETAIL: FIELD \| CONSTR \| METHOD |  |


---


## org.metaqtl Class ChromCluster

```
java.lang.Object
  org.metaqtl.ChromCluster
```

---

``` public class ChromCluster extends java.lang.Object ```

A cluster of chromosomes which share the same name inside a
`MapCluster` framwork.

**Version:**
:   $Version$

**Author:**
:   Jean-Baptiste Veyrieras

**See Also:**: `MapCluster`

---

| **Nested Class Summary** | |
| --- | --- |
| `class` | `ChromCluster.ClusterEdge` |
| `class` | `ChromCluster.ClusterGraph` |


| **Field Summary** | |
| --- | --- |
| `java.util.ArrayList` | `clusters`             The list of the cluster members. |
| `java.util.Hashtable` | `locusNames`             Ths locus name index table |
| `java.lang.String` | `name`             The name of the chromosome cluster. |
| `int` | `ni`             The total number of marker intervals. |
| `int` | `nmc`             Total number of common marker between clusters |


**Constructor Summary**

`ChromCluster()`
  
          Creates a new instance if ChromCluster.


| **Method Summary** | |
| --- | --- |
| `void` | `addChromosome(IBioLGroup group, IBioCross crossDesign, int mappingFunction, int mappingUnit, boolean rescale, boolean skeleton)`             Add a chromosome to the cluster |
| `void` | `fixCluster()`             This methods frozes the index of the distinct markers over all the members of the cluster. |
| `Chromosome[]` | `getClusterMembers()`             This method returns the members of the cluster as an array of `Chromosomes`. |
| `double[]` | `getCMrkTotalNumbers()` |
| `ChromCluster.ClusterGraph` | `getGraph()` |
| `MetaDico` | `getMarkerDico()` |
| `int` | `getMarkerIndexByName(java.lang.String mrkName)`             For a given marker name this method returns the marker index of the marker table if found. |
| `int` | `getMarkerIntervalNumber()`             This method returns the total number of marker intervals, i.e the sum over chromosomes of the number of marker intervals. |
| `java.lang.String` | `getMarkerName(java.lang.String name)`             This method returns the name of the locus from the given name. |
| `java.lang.String` | `getMarkerNameByIndex(int i)`             This method returns the name of the marker according to this indice. |
| `int` | `getMarkerNumber()`             This method returns the number of distinct markers implied in this cluster, i.e the size of the marker table. |
| `int` | `getMarkerOccurence(int i)`             Returns the number of times the marker with the indice i has been seen over the chromosomes of the cluster. |
| `java.lang.String` | `getName()`             Returns the name of the cluster. |
| `boolean` | `isConnected(int nMrk)`             Tests if the cluster of chromosome is connected using a graph approach. |
| `boolean` | `isDubiousLocus(IBioLocus locus)`             If a list of dubious markers has been set before then this routine checks if the given locus is not included into this list. |
| `void` | `setDubiousMarker(IBioLocus[] dubious)`             This method sets the list of dubious marker. |
| `void` | `setMarkerDico(MetaDico mrkDico)` |
| `void` | `setMrkThresh(int mrkThresh)` |

**Methods inherited from class java.lang.Object**

`clone, equals, finalize, getClass, hashCode, notify, notifyAll, toString, wait, wait, wait`

**Field Detail**

### nmc

```
public int nmc
```

:   Total number of common marker between clusters

---


### ni

```
public int ni
```

:   The total number of marker intervals.

---


### name

```
public java.lang.String name
```

:   The name of the chromosome cluster.

---


### locusNames

```
public java.util.Hashtable locusNames
```

:   Ths locus name index table

---


### clusters

```
public java.util.ArrayList clusters
```

:   The list of the cluster members.


**Constructor Detail**

### ChromCluster

```
public ChromCluster()
```

:   Creates a new instance if ChromCluster.


**Method Detail**

### addChromosome

```
public void addChromosome(IBioLGroup group,
                          IBioCross crossDesign,
                          int mappingFunction,
                          int mappingUnit,
                          boolean rescale,
                          boolean skeleton)
```

:   Add a chromosome to the cluster

    :   **Parameters:**: `group` - the chromosome to add.: `crossDesign` - the cross design properties.: `mappingFunction` - the mapping function for this chromosome.: `mappingUnit` - the mapping unit for this chromosome: `rescale` - true if the chromosome must be rescaled @see Chromosome#R2r(): `skeleton` - true if the chromosome if a skeleton chromosome.

---


### isDubiousLocus

```
public boolean isDubiousLocus(IBioLocus locus)
```

:   If a list of dubious markers has been set before
    then this routine checks if the given locus is
    not included into this list.

    :   **Parameters:**: `locus` - the locus to check. **Returns:**: true if the locus is in the dubious marker list, false otherwise. **See Also:**: `setDubiousMarker(IBioLocus[])`

---


### getClusterMembers

```
public Chromosome[] getClusterMembers()
```

:   This method returns the members of the cluster as an
    array of `Chromosomes`. If any chromosome has been
    added to the cluster the method returns null.

    :   **Returns:**: an array of `Chromosomes`.

---


### getMarkerNumber

```
public int getMarkerNumber()
```

:   This method returns the number of distinct markers implied
    in this cluster, i.e the size of the marker table.

    :   **Returns:**: the number of distinct markers.

---


### getMarkerIntervalNumber

```
public int getMarkerIntervalNumber()
```

:   This method returns the total number of marker intervals,
    i.e the sum over chromosomes of the number of marker intervals.

    :   **Returns:**: the total number of marker intervals.

---


### fixCluster

```
public void fixCluster()
```

:   This methods frozes the index of the distinct
    markers over all the members of the cluster.

    :   **See Also:**: `getMarkerNameByIndex(int)`, `getMarkerIndexByName(String)`

---


### getMarkerIndexByName

```
public int getMarkerIndexByName(java.lang.String mrkName)
```

:   For a given marker name this method returns
    the marker index of the marker table if found.
    Otherwise it returns -1.

    :   **Parameters:**: `mrkName` - the name of the marker. **Returns:**: the indice of the marker if found, -1 otherwise. **See Also:**: `fixCluster()`

---


### getMarkerNameByIndex

```
public java.lang.String getMarkerNameByIndex(int i)
```

:   This method returns the name of the marker
    according to this indice. The given indice
    is assumed to be inside the correct range.

    :   **Parameters:**: `i` - the indice of the marker. **Returns:**: the name of the marker. **See Also:**: `fixCluster()`

---


### getMarkerOccurence

```
public int getMarkerOccurence(int i)
```

:   Returns the number of times the marker with
    the indice i has been seen over the
    chromosomes of the cluster.

    :   **Parameters:**: `i` - **Returns:**: **See Also:**: `fixCluster()`

---


### getMarkerName

```
public java.lang.String getMarkerName(java.lang.String name)
```

:   This method returns the name of the locus
    from the given name. If no locus name dictionnary for
    the clusters is defined then the method returns
    an exact copy of the given name.

    :   **Parameters:**: `name` - the raw name of the locus. **Returns:**: the standard name of the locus.

---


### setDubiousMarker

```
public void setDubiousMarker(IBioLocus[] dubious)
```

:   This method sets the list of dubious marker. It must be used
    before adding chromosomes to the chromosome cluster. Then the
    the markers which names matches those of the list will be ignored.

    :   **Parameters:**: `dubious` -

---


### getName

```
public java.lang.String getName()
```

:   Returns the name of the cluster.

    :   **Returns:**: the name of the cluster.

---


### getMarkerDico

```
public MetaDico getMarkerDico()
```

:   **Returns:**: Returns the mrkDico.

---


### setMarkerDico

```
public void setMarkerDico(MetaDico mrkDico)
```

:   **Parameters:**: `mrkDico` - The mrkDico to set.

---


### getCMrkTotalNumbers

```
public double[] getCMrkTotalNumbers()
```

---


### isConnected

```
public boolean isConnected(int nMrk)
```

:   Tests if the cluster of chromosome is connected using a graph
    approach. Each chromosome is related to another by a set of
    common markers. The use can supply a minimal number of common
    marker to declare that a chromosome is connected to the other ones.
    The cluster is said to be connected if there is a path
    connecting every pair of chromosomes.

    :   **Returns:**

---


### setMrkThresh

```
public void setMrkThresh(int mrkThresh)
```

:   **Parameters:**: `mrkThresh` -

---


### getGraph

```
public ChromCluster.ClusterGraph getGraph()
```

:   **Returns:**


---


|  |  |  |
| --- | --- | --- |
| **Overview** \| **Package** \| **Class** \| **Use** \| **Tree** \| **Deprecated** \| **Index** \| **Help** | |  |
| PREV CLASS   **NEXT CLASS** | **FRAMES**    **NO FRAMES**     **All Classes** |  |
| SUMMARY: NESTED \| FIELD \| CONSTR \| METHOD | DETAIL: FIELD \| CONSTR \| METHOD |  |


---
